# Genome-Assisted Characterization of *Lactobacillus fermentum*, *Weissella cibaria,* and *Weissella confusa* Strains Isolated from Sorghum as Starters for Sourdough Fermentation

**DOI:** 10.3390/microorganisms8091388

**Published:** 2020-09-10

**Authors:** Irene Falasconi, Alessandra Fontana, Vania Patrone, Annalisa Rebecchi, Guillermo Duserm Garrido, Laura Principato, Maria Luisa Callegari, Giorgia Spigno, Lorenzo Morelli

**Affiliations:** 1 Department for Sustainable Food Process (DiSTAS), Università Cattolica del Sacro Cuore, 29122 Piacenza, Italy; irene.falasconi@unicatt.it (I.F.); alessandra.fontana@unicatt.it (A.F.); guillermo.dusermgarrido@unicatt.it (G.D.G.); laura.principato@unicatt.it (L.P.); giorgia.spigno@unicatt.it (G.S.); lorenzo.morelli@unicatt.it (L.M.); 2 Biotechnological Research Centre, Università Cattolica del Sacro Cuore, 26100 Cremona, Italy; annalisa.rebecchi@unicatt.it (A.R.); marialuisa.callegari@unicatt.it (M.L.C.)

**Keywords:** sourdough, sorghum flour, starter culture, bakery, lactic acid bacteria

## Abstract

Sourdough fermentation of bakery products is a well-established and widespread technique to confer an added value to the resulting food. In recent decades, gluten-free raw materials have gained more attention due to the diffusion of food disorders such as coeliac disease, but, at the same time, they present difficult manipulation and scarce technological properties because of the absence of gluten. For this reason, the present work was aimed at selecting starter cultures for sourdough application that are isolated from fermentation of sorghum flour. Three isolates of *Lactobacillus fermentum*, *Weissella cibaria,* and *Weissella confusa* were selected for the following properties: exopolysaccharide synthesis, acidification, CO_2_ production, and amylase activity. The investigated phenotypic characteristics were confirmed by genomic analyses, which also highlighted other potentially beneficial features for use in bakery products employment. These strains, together with bakery yeast, were used for bread preparation using sorghum and wheat flour and after 24 h of fermentation the resulting dough was analyzed to assess the improvement of its characteristics. The presence of lactic acid bacteria (LAB) had a great impact on the final dough, and the best preparation, from a rheological point of view, resulted in one made of sorghum and wheat flour with added LAB and bakery yeast, whose resulting characteristics were similar to all wheat flour doughs. The results of this study suggest a potential application of the selected starters in sorghum composite bread and should be validated with data from large-scale pilot tests conducted in industrial bakeries.

## 1. Introduction

It is well established that sourdough bread has a higher nutritional value as well as improved textural and sensory properties compared to yeast-leavened products [1,2,3]. The positive effects of sourdough are mainly due to the metabolic activities of the lactic acid bacteria (LAB) performing the fermentation, which result in increased content of bioactive compounds, increased absorption of vitamins and mineral bioavailability, and decreased glycemic response and antinutritional factor levels [4,5,6,7,8]. Moreover, the activity of LAB improves the bread structure through the production of organic acids and exopolysaccharides (EPS), flavor by the synthesis of volatile compounds, and preservation due to higher acidity, organic acids, and antimicrobial compounds production [9,10,11,12,13,14,15,16,17,18].

Despite the distinctive flavor, taste, and sensory quality of sourdough bread, the use of sourdough fermentation for the manufacture of baked goods poses many challenges for the bakery industry due to the complexity and labor intensity of making and maintaining sourdough through daily refreshments and the long fermentation times required. New biotechnological approaches in sourdough bread production relying on the application of starter cultures have therefore been proposed for the purpose of simplifying and shortening the fermentation process at the industrial level [19,20,21,22]. The proper selection of microorganisms to be used as starter cultures is of paramount importance in sourdough application, since it has a major influence on the characteristics of the final dough, along with the pH and temperature of fermentation. Moreover, several studies have shown that the use of autochthonous LAB to ferment sourdough may represent a valid biotechnology to exploit the potential of non-wheat cereals in breadmaking [23,24,25]. Among these, sorghum has gained increasing attention in developed Western countries in recent years due to its valuable nutritional quality and health-beneficial components. In addition, sorghum is gluten free, making it an attractive alternative food for those who suffer from coeliac disease. A study conducted in 2018 estimated that the global prevalence of coeliac disease varies between 1% and 2% in the general population [26].

Among starter cultures, lactobacilli are the predominant LAB species used, often in mixed cultures including different genera, each with specific desirable properties and functions. Recently, the use of *Weissella* species as novel starter cultures for sourdough bread production has been reported by several authors [27,28,29,30]. The relevance of these heterofermentative LAB is related to their ability to grow at wide temperature, a_w_, and pH ranges [31] and to produce EPS that may improve the rheological properties of dough [32]. Moreover, it was demonstrated that EPS produced in situ, such as dextran, improves the texture and covers unpleasant flavors of wholegrain bread; this can lead to the use of dextran as a possible substitute for masking agents, such as sweeteners [33]. EPS application can also increase crumb softness and delay the staling rate of wheat bread, with a consequent increased shelf-life of the final product [30,34].

The aim of this study was the phenotypic characterization of lactic acid bacteria isolated from spontaneously fermented sorghum flour in order to select potential starters for the production of sorghum-based bread. A genomic analysis was carried out on the most promising candidates to gain insight into the molecular mechanisms underlining desired bacterial features of technological relevance. In addition, rheological analysis of experimental doughs was applied to assess the technological performance of selected strains.

## 2. Materials and Methods

### 2.1. Isolation and Phenotypic Characterization of LAB Isolated from Sorghum Flour

The LAB selected in the present work were isolated by sorghum flour fermentation [35]. After this first fermentation (considered “day 0”) the process was performed following the back-slopping technique, in which ten further refreshments were carried out [36] at a ratio of 1:2:2 (mother dough: sorghum flour: water). Sampling was executed after the spontaneous fermentation (time 0) and then after 1 day, 3 days, 6 days, 8 days, and 10 days during the back-slopping process. For each sampling time, pH, and total titratable acidity (TTA) were determined according to the official AACC method [37]. Aliquots of the sourdough were plated on De Man, Rogosa, Sharpe (MRS, Oxoid™) and modified MRS (mMRS) agar plates for isolating LAB. The mMRS medium was prepared as described by Sekwati-Monang and Gänzle [38]; the composition per liter was as follows: 10.0 g of tryptone, 10.0 g of maltose, 5.0 g of glucose, 5.0 g of fructose, 5.0 g of beef extract, 5.0 g of yeast extract, 4.0 g of potassium phosphate dibasic, 2.6 g of potassium phosphate monobasic, 2.0 g of tri-ammonium citrate, 0.5 g of L-cysteine, 0.2 g of magnesium sulphate, 0.05 g of manganese sulphate, 1.0 g of Tween80, and 15.0 g of bacteriological agar; final pH 6.2. After autoclave sterilization, a 0.002% vitamin solution (B1, B2, B5, B6, B9, and B12) was added by filtration. From the plates of each sample, representative colonies were selected based on color, size, and morphology. DNA was extracted from isolated purified colonies using microLYSIS® (Microzone, Brighton, UK) following the manufacturer’s protocol. The isolates were screened by repetitive element palindromic polymerase chain reaction (REP-PCR) using the single stranded oligonucleotide primer GTG-5 (5′-GTG GTG GTG GTG GTG-3′) [39]. One representative for each cluster was identified by amplifying the 16S *rRNA* gene using the primers P0 (5′-GAG AGT TTG ATC CTG GCT-3′) and P6 (5′-CTA CGG CTA CCT TGT TAC-3′) [40]. The PCR products were purified using the MicroCLEAN PCR Clean-Up system (Microzone) and sequenced at the BMR Genomics facility (Padova, Italy). The identification of sequences was performed by alignment against the Ribosomal Database Project (RDP) database using the naïve Bayesian classifier [41].

Potential starter cultures were then selected according to four main features: acidification activity [42], amylolytic activity [43], EPS production [44], and gas production using Durham tubes inserted into tubes containing mMRS liquid medium. These phenotypic traits are considered among the main desirable characteristics for the selection of starters to be employed in bakery products, as previously reported [45].

### 2.2. Safety of the Selected Isolates

The susceptibility profiles of the selected strains to ampicillin, gentamycin, kanamycin, streptomycin, neomycin, tetracycline, erythromycin, clindamycin, and chloramphenicol were determined by broth microdilution following the ISO 10932/IDF223 protocol [46]. Results were compared to the cut-off values proposed by the European Food Safety Authority (EFSA) [47]. Since EFSA does not provide breakpoints for *Weissella* spp., for these isolates the cut-off values of obligate heterofermentative lactobacilli were considered.

In order to confirm the susceptibility profiles obtained by phenotypic analyses, PCR for detecting antibiotic resistance genes was performed. In particular, three aminoglycoside-modifying enzyme genes were tested: *aph(3′)-III* [48], *aac(6′),* and *ant(4′)* [49].

Furthermore, the production of biogenic amines was assessed by following the protocol of Bover-Cid and Holzapfel [50].

### 2.3. Whole-Genome Sequencing and Bioinformatic Analyses

Genomic DNA of the three strains in the study was extracted using the MasterPure™ Gram Positive DNA Purification Kit (Lucigen Corporation, Middleton, WI, USA), according to the provided protocol. The quality of the extracted DNA was then checked by agarose gel electrophoresis (0.8%) and the quantity was determined using a Qubit fluorometer (Life Technologies, Carlsbad, CA, USA).

Genomic DNA was shotgun sequenced on the Illumina NextSeq platform, using the NextSeq High-Output kit (Illumina Inc., San Diego, CA) for library preparation (150 + 150 bp). Quality filtering and adaptors removal were carried out using Trimmomatic software (v0.32) [51].

De novo genome assemblies were obtained with the Shovill pipeline version 0.9 (https://github.com/tseemann/shovill) and assembled with SPAdes 3.12.0 [52] using k-mer sizes 31, 51, 71, 91, and 111, under default parameters. The quality of the assembled genomes was evaluated with QUAST (v4.6.0) [53]. Assemblies annotation was performed by comparing RAST and Prokka (v1.13.3) tools [54,55].

The presence of complete prophage regions and antibiotic resistance genes in the genomic sequences was evaluated by means of PHASTER and the resistance gene identifier (RGI) [56,57]. The identification of bacteriocin sequences was done using BAGEL4 [58], whereas the detection of CRISPRs and *cas* genes was carried out with CRISPRCasFinder [59]. The presence of plasmids was evaluated with PlasmidFinder 2.1 [60].

### 2.4. Dough Preparation

The dough was prepared following a basic recipe, as follows: 495 g of wheat Manitoba flour type “0” (Farina d’America, Molino Spadoni, Italy), 5 g of white sugar (Zefiro, Eridania, Italy), 50 g of extra virgin olive oil (100% Italian, Coop, Italy), 250 mL of tap water, and 5 g of salt. The kneading process was done using an electric home kneading machine (IMETEC ZERO-GLU KM 1500) initially with all the ingredients except salt, at machine speed 1 for 5 min. Then salt was added, and the mixture was kneaded for a further 5 min at speed 1 and 4 min at speed 2. Sugar was added to enhance yeast activity and EPS production by LAB. White sorghum flour (Farina di sorgo bianco, Mulino Marello, Italy) was used alone (recipe of 100% sorghum) or at a 1:1 ratio with wheat Manitoba flour type “0” (recipe of 50% sorghum). Each recipe was tested with three different inocula: fresh commercial bakery yeast alone (Lievito fresco, Conad, Italy); LAB isolated from sorghum alone; and a combination of yeast and LAB.

For the yeast-only inoculum, the commercial yeast was added at a concentration of 10^6^ CFU/g dough. For the LAB inoculum, three bacteria were selected according to their phenotypic characteristics: *Weissella cibaria* UC4051, *Weissella confusa* UC4052, and *Lactobacillus fermentum* UC3641. The LAB were cultivated in MRS at 30 °C overnight. Cells were harvested by centrifugation (3500 × *g*, 10 min), washed in saline solution twice and added in a final concentration of 10^7^ CFU/g dough [61]. The viability of cells (CFU/mL) was tested by an MRS agar plate count, after incubation at 30 °C for 48 h under anaerobic conditions. Additionally, the recipes with 100% and 50% sorghum with both yeast and LAB were also tested with a higher sugar concentration (10 wt % of flour weight) in order to evaluate if such a high concentration could increase EPS production and have an effect on the rheological property of the dough.

The final dough of each recipe was divided into four loaves of fixed dimensions (2.5 cm height and 7 cm diameter) and weight (around 120–125 g). Leavening was carried out at 26 °C at 82% of relative humidity for 24 h in a climatic chamber (Memmert, Schwabach, Germany).

### 2.5. Technological Characterization of the Dough

Each loaf was analyzed for the following parameters: yeast count, LAB count, pH (pHmeter Hanna Edge), a_w_ (Aqualab Serie 4; Steroglass, Perugia, Italy), height, diameter, weight, and rheological characteristics before (time 0) and after fermentation (time 24).

Yeast and LAB counts were performed in duplicate and Rose Bengal (RB) agar was added with chloramphenicol and MRS agar with 1% of cycloheximide, respectively.

The rheological characteristics of the dough samples were evaluated through rheological tests carried out in the oscillatory mode. Tests were carried out at 30 ± 0.1 °C in an Anton Paar MCR 302 rheometer (Anton Paar, Austria) with a knurled plate-flat geometry (25 mm diameter, PP25/P2—SN13968) equipped with a Peltier system for temperature control. The dough was placed between the plates and the gap adjusted to 3.5 mm; the dough’s edges were trimmed and immediately coated with mineral oil to prevent drying. A resting time of 5 min was set for relaxation of the doughs before performing the amplitude and frequency sweep tests.

An amplitude sweep test was conducted to determine linear viscoelastic region (LVR) using an amplitude strain from γ = 0.01 to 100 s^−1^ at a constant frequency of 1 Hz. Then, frequency sweep tests were performed at a constant shear strain of 0.01% (within the LVR region obtained from the previous amplitude sweep test) and varying oscillation frequency between 0.1 and 100 Hz. This is because the fundamental rheological parameters calculated by the instrument software require validation that the samples are linearly viscoelastic and because linear behavior under low strains implies that the small deformation is not injurious to the dough’s structure [62]. The obtained parameters are the elastic (storage modulus or G′) and viscous (loss modulus or G”) components of a complex viscosity η*, which is calculated according to (1):

(1) $$\eta^{*}=\frac{\sqrt{\left(G^{\prime 2}+G^{\prime \prime 2}\right)}}{\omega }$$

### 2.6. Statistical Analysis

The influence of the inoculum type on the physical–chemical characteristics of the dough was assessed by one-way ANOVA (analysis of variance) at a confidence level of 95% (*p* < 0.05). In case of significant influence, the Tukey’s post-hoc test was applied for mean discrimination, always at a 95% confidence level. Statistical software SPSS^®^ (version 21.0, SPSS Inc., Chicago, IL, USA) was used.

## 3. Results and Discussion

### 3.1. Phenotypic Characterization of LAB Isolated from Sorghum Flour

**Figure 1:**
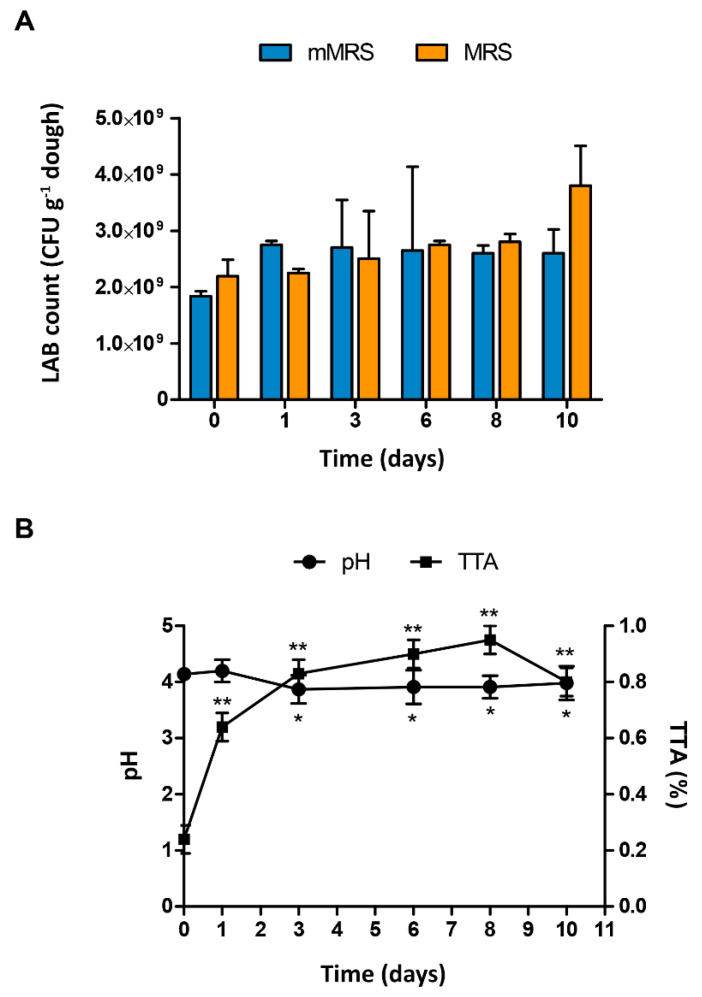
(**A**) Counts of lactic acid bacteria (LAB) in De Man, Rogosa, Sharpe (MRS) and modified MRS (mMRS) during spontaneous fermentation of sorghum flour sourdoughs for 10 days. (**B**) Total titratable acidity (TTA) values during spontaneous fermentation. One-way ANOVA analysis followed by a Tukey’s test was made to compare all the values to the ones of time 0; asterisks indicate significant (*p* < 0.05) differences.

The course of the total LAB counts in mMRS and MRS, as well as the pH and TTA during sorghum sourdough fermentation, are shown in Figure 1.

A total of 178 isolates were selected at different time points across the back-slopping process. The comparison of all REP banding patterns allowed identification of 20 different profiles. A representative isolate for each REP profile was identified by 16S *rRNA* gene analysis and tested for the phenotypic characteristics listed above. Table S1 shows the different species isolated during fermentation. A prevalence of *Weissella* spp. was detected in the early stages of the process, while at the end of the fermentation *Pediococcus* spp. and *L. fermentum* were predominant.

A summary of all the phenotypic characteristics of the isolates is shown in Table 1. The group having the best acidification activity was constituted by *Pediococcus* spp., with *P. pentosaceus* P3 being able to reach a final pH of 3.31, and with a reduction of 2.8 pH points after 72 h of incubation. Carbon dioxide production was observed in all the strains except *Pediococcus* spp., as expected; among gas producers, the best performer was *L. fermentum* UC3641, since it was able to fill the Durham tube with its CO_2_ production.

On the other hand, amylolytic activity was detected only in *W. cibaria* UC4051 and *L. fermentum* UC3641. Finally, EPS production assessment revealed that all of the *W. cibaria* and *W. confusa* strains were able to produce exopolysaccharides starting from sucrose, and *W. confusa* UC4052 seemed to show the stronger mucoid phenotype among all the producers; indeed, it was the only isolate where EPS production overwhelmed the streaked-out area, making separate streaks hard to see. This is in line with previous studies, which reported *W. confusa* as a high EPS producer [63]. In summary, the selected strains were: *W. cibaria* UC4051 for its amylolytic activity, *W. confusa* UC4052 for being the major EPS producer, and *L. fermentum* UC3641 for its gas production and acidification activity.

### 3.2. Safety of the Selected Isolates

The results of the antibiotic resistance assessment together with the EFSA breakpoints are reported in Table S2. All strains are demonstrated having MICs lower than the stated breakpoints, except for kanamycin: *W. cibaria* showed a MIC value of 64 µg/mL, while the EFSA breakpoint was 32 µg/mL. Nevertheless, none of the selected genes encoding for enzymes that confer aminoglycosides resistance were detected in our strains.

The aminogenic potential of the investigated strains was negative. This is in line with previous studies. Even if LAB are considered the main biogenic amine producers in fermented food, the genera *Weissella* and *Leuconostoc* were demonstrated to be minor producers, as *L. fermentum* is not the major producer among the *Lactobacillus* genus [64].

### 3.3. General Genomic Features of Candidate Starter Cultures

The main characteristics of the three genomes sequenced are reported in Table S3. Both species of *Weissella* were similar in genome size (2.44 Mbp and 2.33 Mbp for *W. cibaria* UC4051 and *W. confusa* UC4052, respectively) and GC content (45%), whereas *L. fermentum* UC3641 presented a smaller genome (1.97 Mbp) and a higher GC content (52%). Plasmids were absent in all genomes. On the other hand, complete prophage regions were identified in both *Weissella* species (one for *W. cibaria* UC4051 and two for *W. confusa* UC4052). CRISPR/Cas system-related sequences were found in *L. fermentum* UC3641, namely two CRISPRs and seven associated proteins (Cas1, Cas2, Cas3, Cas6, Cas9, CasC, and Cse3). Concerning mobile elements, 10 transposases belonging to five IS families (IS3, IS6, IS30, IS1182, and ISL3) were present in *W. cibaria*, whereas 20 transposases belonging to seven IS families (IS3, IS4, IS5, IS30, IS256, IS200/IS605, and ISL3) were identified in *L. fermentum*. However, no IS elements were found in *W. confusa*, which could indicate a higher stability of its genetic content. Indeed, in bacteria, mobile genetic elements, which include integrases and insertion sequences, are primary contributing factors to genetic diversity and niche adaptation [65].

It is important to highlight that no antibiotic resistance determinants were detected in all three genomes, confirming the phenotypic results.

#### 3.3.1. Carbohydrate Metabolism

**Figure 2:**
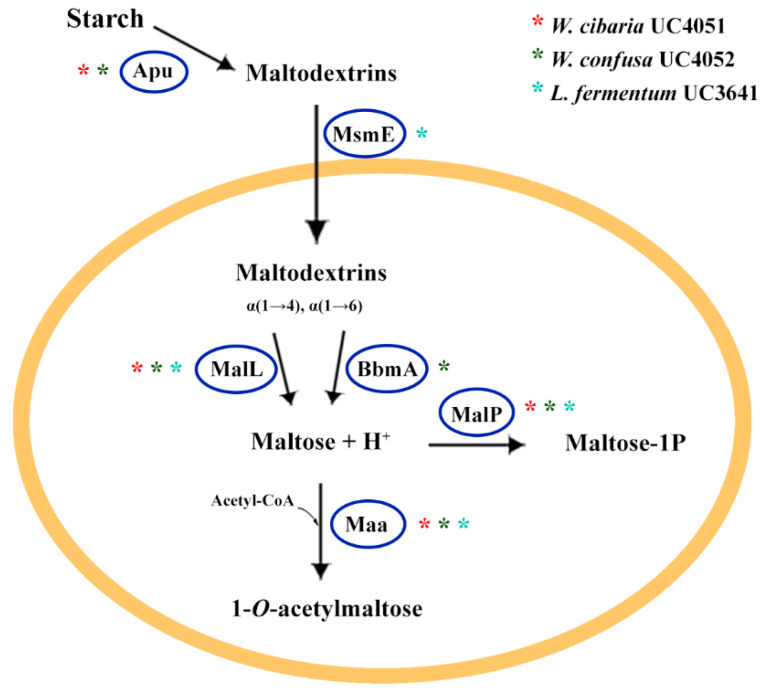
Schematic representation describing starch degradation operated by the three strains selected as starters for sorghum sourdough fermentation.

From the RAST subsystem category related to carbohydrate metabolism, it was shown that both *W. cibaria* UC4051 and *W. confusa* UC4052 could use mannose, D-ribose, xylose, D-gluconate, and ketogluconates as monosaccharides. Interestingly, only *W. cibaria* contains genes for the utilization of L-arabinose, D-galacturonate, and D-glucuronate metabolisms. In addition, *L. fermentum* UC3641 can also use D-galactarate, D-glucarate, and D-glycerate as monomeric sugars. Concerning di- and oligo-saccharides, all the strains can utilize sucrose, lactose, and galactose, but *L. fermentum* can also metabolize fructooligosaccharides (FOS) and raffinose. In relation to starch degradation, gene annotation by Prokka highlighted the presence of an extracellular amylopullulanase (*apu*) in the two *Weissella* genomes and an intracellular maltogenic amylase (*bbmA*) in *W. confusa* (Figure 2). Moreover, an intracellular glucosidase (*malL*), which hydrolyzes α(1→6)-glucosidic linkages, a maltose phosphorylase (*malP*), epimerase, and O-acetyltransferase (*maa*) were found in all strains. A putative maltose binding protein (MsmE) was also identified in *L. fermentum* (Figure 2). Four gene copies of 6-phospho-β-glucosidase (*bglA*) were detected in both *Weissella* species, one copy of *bglB* was found in *W. cibaria* and *L. fermentum*, whereas *bglH* was found in four and two copies in *W. cibaria* and *W. confusa*, respectively. These enzymes belong to the glycoside hydrolase family 1 (GH-1) and catalyzes the hydrolysis of β(1→4)-glucosidic linkages.

Metabolic conversion of maltose and sucrose contributes to the final texture of the dough since EPS can be eventually produced. Moreover, the presence of EPS in sourdough increases bread volume, along with the content of dietary fibers [3]. From RAST annotation, genes belonging to the EPS cluster (i.e., *epsB*, *epsC*, *epsD*, and *epsE*) were present in *W. cibaria* and *L. fermentum*; Prokka also identified the genes *epsF* and *epsH*. Differently from the phenotypic results, *W. confusa* did not present an EPS cluster. However, this species possesses three genes coding for poly-β-1,6-N-acetyl-D-glucosamine synthase (one copy of *pgaC* and two copies of *icaA*), which has been reported to be a surface polysaccharide involved in biofilm formation [66]. This could be related to the higher production of EPS found in *W. confusa*, since *W. cibaria* presents only one copy of the *pgaC* gene. Additionally, capsular polysaccharide phosphotransferase genes were detected in both *Weissella* species, namely *cpsY* and *cps12A* in *W. cibaria* and *W. confusa*, respectively. Multiple copies of glycosyltransferases belonging to the GH70 family were also detected (*gtf1* and *gtfC*), putatively involved in EPS production.

#### 3.3.2. Protein and Amino Acid Metabolism

Peptides and amino acid metabolites influence the final food product as taste-active compounds, flavor precursors, or bioactive molecules [67]. All the strains showed an almost complete Pep system, including *pepA*, *pepC*, *pepD*, *pepF*, *pepI*, *pepN*, *pepO*, *pepQ*, *pepS*, *pepT*, *pepV*, and *pepX* genes. *L. fermentum* UC3641 contains three copies of the *pepD* gene, whose main target is dipeptides. ATP-dependent protease genes are also present in all the genomes, specifically *clpC* (two copies), *clpE*, *clpP* (two copies in both *Weissella* spp.), and *clpX*, along with two putative heat shock protease genes (*htrA* and *htpX*).

Concerning the transport mechanisms, *L. fermentum* UC3641 seems to include only di-/tripeptide transporters (two copies of the *dtpT* gene), whereas in the *Weissella* species, a complete Opp system was found, dedicated to oligopeptides transportation (i.e., *oppA*, *oppB*, *oppC*, *oppD*, and *oppF* genes), together with the *dtpT* gene and the di-/tripeptide permease genes *dtpA* and *dppC*.

Interestingly, a complete arginine deiminase (ADI) pathway was detected in all strains, including arginine deiminase (ArcA), ornithine carbamoyltransferase (ArcB), and carbamate kinase (ArcC). Moreover, all genomes also possess the dedicated arginine/ornithine antiporter (ArcD), which is present in double copy in *L. fermentum* UC3641. The release of ornithine via the ADI pathway is considered very important, since this compound is one of the key flavor precursors in sourdough. Indeed, it has been reported that ornithine reacts with carbohydrates during the baking process, generating 2-acetyl-l-pyrroline, a precursor for flavor compounds in the wheat bread crust [68]. Furthermore, the *L. fermentum* strain seemed to be able to produce other flavor active compounds derived from phenylalanine metabolism [68]. It indeed presented the genes coding for phenylacetaldehyde, phenylacetate, and phenylethanol.

#### 3.3.3. Bioactive Compounds

*W. cibaria* UC4051 included complete pathways for the putative synthesis of B vitamins, namely folate (B9), thiamine (B1), riboflavin (B2), pyridoxine (B6), nicotinate, and nicotinamide. The production of such compounds during sourdough fermentation suggests a health-modulating potential of these strains if utilized as sourdough starters. Moreover, *L. fermentum* UC3641 presented a gene implied in cinnamic acid metabolism (i.e., cinnamic acid-dihydrodiol dehydrogenase, *hcaB*), the degradation of which, as a phenolic compound, influences the final aroma and the release of antioxidant molecules [69]. Furthermore, three genes encoding peroxidases, namely NADH peroxidase (*npr*), alkyl hydroperoxide reductase (*ahpC*), and thiol peroxidase (*tpx*), were also highlighted in *L. fermentum*. These proteins have an important role by acting as antioxidants [70]. Among these genes, the two *Weissella* species possess only *npr*, with *W. cibaria* showing two copies.

Interestingly, all the strains include phosphatases related to the inositol-phosphate metabolism. Specifically, *W. confusa* UC4052 presents three dedicated enzymes, whereas *W. cibaria* UC4051 and *L. fermentum* UC3641 exhibit five enzymes, indicating the ability to metabolize phytate derivatives. Phytases are myo-inositol phosphohydrolase, which catalyze the hydrolysis of phytic acid to myo-inositol and phosphoric acid. These enzymes produce available phosphate along with non-metal chelator compound and are considered of great value to improve the nutritional quality of phytate-rich foods [71].

Regarding defense mechanisms, only one gene coding for a bacteriocin (i.e., Enterolysin A) was found in *L. fermentum* UC3641. Although the presence of the gene does not imply the production of the protein, this potential production is important for its possible implication in preventing the growth of undesired bacteria in the final product, enhancing its quality and safety profile.

### 3.4. Technological Properties of the Dough

The results for a_w_, weight, height, diameter, and total yeast and LAB counts after fermentation for the different tested recipes are shown in Table 2 and Table 3.

Considering the base recipe with 1% sugar (Table 2), the acidifying effect of the LAB starter leading to a significant reduction of pH, both when used alone and in combination with bakery yeast (around pH 4.2) and independently on the sorghum flour percentage, is statistically significant, as expected. This agrees with the LAB count after 24 h of fermentation, which was the same for all the recipes with LAB addition as starters or costarters.

Moreover, in both recipes, water activity was lower with the addition of only LAB with respect to the only-yeast inoculum; this suggests that the EPS and lactic acid produced by LAB retain water, leading to a lower a_w_ and weight loss [72,73].

Considering the spread factor, the best combination of dough is that made with 1% sugar with the addition of yeast and LAB.

The addition of sugar at 10% of flour weight (Table 3) slightly reduced the a_w_ and increased the pH compared to the control with 1%, without substantial effects on yeast and LAB counts. Nonetheless, the spread factor in the recipe with wheat flour increased after 24 h, due to a significant increase of the loaves’ diameter, while in the 100% sorghum flour recipe the spread factor remained almost the same, meaning that the addition of only our starters is not sufficient to raise the volume of the loaves, due to the absence of gluten that entraps the CO_2_ produced by the microorganisms. It is likely that modifying the recipe to include a supplement of corn [74] or potato [75] starch could improve the leavening of the gluten-free dough.

Regarding the rheological characterization of the doughs, for all the samples the G′ (elastic component) was greater than the G” (viscous component), behavior typical of an elastoviscous solid-like body. Weipert [76] reports that resistant and poorly extensible doughs typically show high G′ and low G” with consequent low values of the loss tangent due to the high difference between the two modules. On the other hand, doughs with low G′ and G” values indicate greater extensibility and, for gluten-containing flours, fewer starch–gluten interactions.

In our samples, we always observed a smaller loss tangent reflecting a more rigid and stiff material due to the sorghum flour addition. In all the doughs, the log of G′ and G” linearly increased with increasing log of frequency. The loss factor was in the range 0.17–0.35 for all the doughs prepared with 100% sorghum, while generally higher and in the range 0.3–0.7 for the doughs with 50% sorghum, confirming that gluten presence allows for greater extensibility. Moreover, looking at the values of G′ and G”, these were always definitely too high for the doughs with 100% sorghum, as well as the complex viscosity after fermentation. Those results are typical for gluten-free bread. In their study, Ahmed et al. [77] reported that the addition of lupine flour caused a shift of curves G′ and G” towards higher values, while curve tan δ moved towards lower values. Likewise, Lamacchia et al. [78] recorded significantly higher values of G′ and G” for oat whole meal dough than for wheat dough. Finally, Monthe at al. [79] found an optimal value of G′ between 14,000 and 16,000 Pa for prefermented sorghum/sweet potato/cassava starch blend.

After the 24 h leavening, we observed a different behavior depending on the used inoculum and sorghum percentage. Doughs with an elastoviscous solid-like body follow a power law model (2) and (3) that regulates the flow-properties throughout the rheological parameters ${G^{\prime }}_{0}$ and ${G^{\prime \prime }}_{0}$ (Pa) and n (dimensionless).

(2) $$G^{\prime }={G^{\prime }}_{0}\omega^{n}$$

(3) $$G^{\prime \prime }={G^{\prime \prime }}_{0}\omega^{n}$$

It was verified that experimental data followed the power law models and the main differences were detected in $G^{\prime }$ trends, which are reported in Table 4.

For the reference doughs of 100% sorghum prepared with only bakery yeast, there was a slight increase in both the elastic and viscous modulus and, consequently, in the complex viscosity. On the other hand, when LAB were used alone, in the 100% sorghum doughs there was a slight reduction (around 10%) in complex viscosity due to a similar reduction in the storage modulus G′, which expressed tenacity and elasticity, while the loss modulus G″ (extensibility) remained almost constant. A more remarkable effect was detected when LAB were used in combination with yeast, where G′ was reduced about one third of the initial value. For the 50% sorghum samples, the complex viscosity after both yeast and LAB fermentation was 50–60% lower than the initial dough due to a reduction of the G′ modulus. When LAB were used in combination with yeast, we observed almost the same reduction seen for the sample of 100% sorghum, obtaining values after fermentation more similar to the yeast control.

According to those latter results, samples of 50% and 100% were reprepared, increasing the percentage of sugar from 1% to 10%. The data are reported in Table 4. No improvement was detected for the sample of 100% sorghum. However, the dough with 50% showed an even lower value of G_0_′ than the control and was more similar to wheat flour (n = 0.33 and G_0_′ = 3000 Pa), which could be due to a synergic effect between LAB and yeast and a higher production of exopolysaccharides.

## 4. Conclusions

In this study, we selected three strains as starters for sorghum sourdough fermentation. Specifically, *W. cibaria* UC4051 was chosen for its amylolytic activity, *W. confusa* UC4052 for being the major EPS producer among the isolated microorganisms, and *L. fermentum* UC3641 for its gas production and acidification activity. These autochthonous strains were demonstrated as being suitable for use in breadmaking since they were seen to explicate well all the features for which they were selected. Furthermore, they are safe according to the EFSA breakpoints. These starter cultures together with bakery yeast exhibited an improvement of the rheological and textural characteristics of the sourdough. Being naturally adapted to the characteristics of the raw material, such strains are likely to perform better in sourdough fermentation of sorghum bread as compared to conventional starters isolated from different sources. The most beneficial effect can be observed in the recipe with 50% sorghum flour, 50% wheat flour, and 10% sucrose, and it could be mainly due to the increased production of EPS and organic acids by LAB, along with the presence of gluten in the wheat flour.

Further studies will be needed to improve their use in gluten-free doughs and should consider changes in the recipe to include other ingredients, which could enhance the leavening and the promoting effects of these starter cultures.

## Figures and Tables

**Table 1:** Results of the phenotypic characterization of LAB isolated from sorghum sourdough.

Microorganism	Acidification (Final pH)	CO_2_ Production ^1^	Amylolytic Activity	EPS Production ^2^
*W. paramesenteroides* W1	3.99 ± 0.01	+	−	−
*W. cibaria* W2 (UC4051)	3.84 ± 0.04	+	+	+
*W. confusa* W3	3.67 ± 0.03	+++	−	+
*W. confusa* W4	3.66 ± 0.01	+	−	+
*W. cibaria* W5	3.98 ± 0.21	+	−	+
*W. paramesenteroides* W6	3.92 ± 0.13	+	−	−
*W. confusa* W7	4.08 ± 0.03	++	−	+
*W. confusa* W8 (UC4052)	3.80 ± 0.06	+	−	++
*W. cibaria* W9	3.73 ± 0.04	++	−	+
*W. confusa* W10	3.72 ± 0.14	+	−	+
*P. pentasaceus* P1	3.64 ± 0.00	−	−	−
*P. pentasaceus* P2	3.32 ± 0.04	−	−	−
*P. pentasaceus* P3	3.31 ± 0.02	−	−	−
*P. pentasaceus* P4	3.35 ± 0.28	−	−	−
*P. pentasaceus* P5	3.77 ± 0.08	−	−	−
*P. pentasaceus* P6	3.51 ± 0.11	−	−	−
*P. acidilactici* P7	3.71 ± 0.06	−	−	−
*L. fermentum* L1	4.03 ± 0.13	+	−	−
*L. fermentum* L2 (UC3641)	3.75 ± 0.12	+++	+	−
*Lc. taiwanensis* L3	3.81 ± 0.21	−	−	−

^1^ CO_2_ production: − no gas production; + a small bubble was detected in the Durham tube; ++ half of the Durham tube was occupied by gas; +++ all the Durham tube was full of gas. ^2^ EPS production: - growth shows no evidence of EPS production; + both the confluent area and the streaked-out region are mucoid in appearance and the growth is flat; and ++ EPS production overwhelms the streaked-out area, making separate streaks hard to see.

**Table 2:** Summary of the parameters measured after 24 h of incubation of the dough containing different concentrations of sorghum flour.

Parameter	Yeast	LAB	Yeast + LAB
**50% Sorghum ^1^**			
pH	5.528 ± 0.024 ^a^	4.202 ± 0.004 ^b^	4.178 ± 0.008 ^c^
a_w_	0.9743 ± 0.0008 ^a^	0.9701 ± 0.0053 ^b^	0.9629 ± 0.0008 ^c^
Weight (g)	113.05 ± 0.61 ^a^	113.98 ± 0.42 ^b^	114.79 ± 0.42 ^c^
Height (H) (cm)	3.43 ± 0.17 ^a^	3.10 ± 0.17 ^b^	3.45 ± 0.21 ^a^
Diameter (D) (cm)	9.13 ± 0.21 ^a^	9.25 ± 0.29 ^a^	9.00 ± 0.00 ^a^
Spread factor (D/H)	2.66 ± 0.15 ^a^	2.98 ± 0.19 ^b^	2.61 ± 0.16 ^a^
LAB Log (CFU/g)	7.10 ± 0.04 ^a^	9.16 ± 0.06 ^b^	9.25 ± 0.07 ^b^
Yeast Log (CFU/g)	7.59 ± 0.01 ^a^	4.62 ± 0.12 ^b^	5.77 ± 0.10 ^c^
**100 % Sorghum ^2^**			
pH	5.406 ± 0.011 ^a^	4.298 ± 0.025 ^b^	4.356 ± 0.040 ^c^
a_w_	0.9752 ± 0.0016 ^ab^	0.9713 ± 0.0043 ^b^	0.9792 ± 0.0038 ^a^
Weight (g)	114.67 ± 0.61 ^a^	113.08 ± 0.86 ^b^	110.55 ± 0.23 ^c^
Height (H) (cm)	2.75 ± 0.17 ^a^	2.75 ± 0.13 ^a^	2.80 ± 0.18 ^a^
Diameter (D) (cm)	6.88 ± 0.05 ^a^	7.13 ± 0.10 ^b^	7.15 ± 0.06 ^b^
Spread factor (D/H)	2.50 ± 0.16 ^a^	2.59 ± 0.13 ^a^	2.55 ± 0.17 ^a^
LAB Log (CFU/g)	7.62 ± 0.01 ^a^	9.31 ± 0.11 ^b^	9.42 ± 0.08 ^b^
Yeast Log (CFU/g)	7.37 ± 0.10 ^a^	5.49 ± 0.15 ^b^	6.13 ± 0.02 ^c^

^1^ Dough containing 50% sorghum flour 50% wheat flour and 1% sugar concentration. ^2^ Dough containing 100% sorghum flour and 1% sugar concentration. Data are expressed by mean values ± SD. In each row, values not sharing a common superscript letter are significantly different according to the Tukey’s test for comparison of means (*p* < 0.05).

**Table 3:** Results of the dough made with yeast and LAB, sorghum flour, and sucrose concentrations.

	T0 ^1^	T24	T24
Parameter		1% Sugar	10% Sugar
**50% Sorghum ^2^**			
pH	5.876 ± 0.006 ^a^	4.166 ± 0.006 ^b^	4.356 ± 0.006 ^c^
a_w_	0.9795 ± 0.0107 ^a^	0.9717 ± 0.0018 ^b^	0.9676 ± 0.0023 ^b^
Weight (g)	120.23 ± 0.13 ^a^	112.53 ± 0.24 ^b^	112.48 ± 0.31 ^b^
Height (H) (cm)	2.87 ± 0.16 ^a^	3.28 ± 0.22 ^b^	2.89 ± 0.24 ^b^
Diameter (D) (cm)	6.90 ± 0.07 ^a^	9.15 ± 0.58 ^b^	9.70 ± 0.26 ^b^
Spread factor (D/H)	2.40 ± 0.14 ^a^	2.79 ± 0.26 ^b^	3.43 ± 0.29 ^c^
LAB Log (CFU/g)	7.16 ± 0.04 ^a^	9.46 ± 0.06 ^b^	9.16 ± 0.02 ^c^
Yeast Log (CFU/g)	6.19 ± 0.06 ^a^	6.59 ± 0.10 ^a^	6.50 ± 0.28 ^a^
**100% Sorghum**			
pH	5.930 ± 0.054 ^a^	4.546 ± 0.011 ^b^	4.684 ± 0.018 ^c^
a_w_	0.9760 ± 0.0098 ^a^	0.9704 ± 0.0020 ^b^	0.9626 ± 0.0010 ^c^
Weight (g)	120.40 ± 0.19 ^a^	113.91 ± 0.30 ^b^	114.12 ± 0.30 ^b^
Height (H) (cm)	2.75 ± 0.07 ^a^	2.90 ± 0.00 ^b^	2.98 ± 0.05 ^b^
Diameter (D) (cm)	6.66 ± 0.03 ^a^	6.50 ± 0.10 ^a^	6.65 ± 0.13 ^a^
Spread factor (D/H)	2.43 ± 0.06 ^a^	2.24 ± 0.10 ^b^	2.23 ± 0.06 ^b^
LAB Log (CFU/g)	7.78 ± 0.01 ^a^	9.39 ± 0.02 ^b^	9.17 ± 0.07 ^b^
Yeast Log (CFU/g)	6.15 ± 0.02 ^a^	5.71 ± 0.16 ^b^	5.61 ± 0.19 ^b^

^1^ The reported T0 is a mean of all the values of the two doughs before incubation. ^2^ Dough containing 50% of sorghum flour and 50% of wheat flour. Data are shown as means ± SD. In each row, numbers not sharing a common superscript letter are significantly different according to the Tukey’s test for comparison of means (*p* < 0.05).

**Table 4:** Linear regression parameters (n and ${G^{\prime }}_{0}$ ) from application of the power law model (2) of elastic modulus $G^{\prime }$ curves. R^2^: linear regression coefficient.

	t_0_			t_24_		
Dough	n (−)	G_0′_ (Pa)	R^2^	n (−)	G_0′_ (Pa)	R^2^
100% Sorghum + Yeast	0.118	158,800	0.992	0.103	166,809	0.996
100% Sorghum + LAB	0.107	178,813	0.996	0.114	157,217	0.999
100% Sorghum + Yeast + LAB	0.113	149,142	0.994	0.120	54,790	0.994
50% Sorghum + Yeast	0.160	17,128	0.997	0.283	5,857	0.987
50% Sorghum + LAB	0.158	28,474	0.989	0.187	17,689	0.997
50% Sorghum + Yeast + LAB	0.175	21,535	0.993	0.241	12,405	0.984
100% Sorghum + 10% Sucrose + Yeast + LAB	0.100	119,729	0.992	0.126	104,761	0.999
100% Sorghum + 1% Sucrose + Yeast + LAB	0.113	149,142	0.994	0.120	54,790	0.994
50% Sorghum + 10% Sucrose + Yeast + LAB	0.160	12515	0.994	0.316	4688	0.981
50% Sorghum + 1% Sucrose + Yeast + LAB	0.176	24949	0.993	0.251	8054	0.981

## Data Availability

Sequence data used in this study were deposited at the National Center for Biotechnology Information (NCBI) as part of the BioProject PRJNA524466. Whole Genome Shotgun assemblies have been deposited at DDBJ/ENA/GenBank under the accessions SJJV00000000 (*Lactobacillus fermentum* UC3641), SJJX00000000 (*Weissella cibaria* UC4051), and SJJW00000000 (*Weissella confusa* UC4052).

## Supplementary Materials

The following are available online at https://www.mdpi.com/2076-2607/8/9/1388/s1, Table S1: Lactic acid bacteria detected during the back-slopping procedure in sorghum sourdoughs. In brackets, the number of strains isolated for each species; Table S2: MIC (µg/mL) of antibiotics exhibited by the strains isolated in this study; Table S3: General features of the three sequenced genomes, *W. cibaria* UC4051, *W. confusa* UC4052, and *L. fermentum* UC3641, isolated from sorghum sourdough.
