# Supplementary material for: Genome-Assisted Characterization of Lactobacillus fermentum, Weissella cibaria, and Weissella confusa Strains Isolated from Sorghum as Starters for Sourdough Fermentation
Source: Microorganisms. 2020 Sep 10;8(9):1388. doi: 10.3390/microorganisms8091388 (PMC7565839; doi:10.3390/microorganisms8091388)
Supplement: Supplementary file 1 [file microorganisms-08-01388-s001.pdf]

Article

Genome-Assisted Characterisation of *Lactobacillus fermentum, Weissella cibaria,* and *Weissella confusa* Strains Isolated from Sorghum as Starters for Sourdough Fermentation

Irene Falasconi [1,†], Alessandra Fontana [1,†], Vania Patrone [1,*], Annalisa Rebecchi [2], Guillermo Duserm Garrido [1], Laura Principato [1], Maria Luisa Callegari [2], Giorgia Spigno [1] and Lorenzo Morelli [1]

[1] Department for Sustainable Food Process (DiSTAS), Università Cattolica del Sacro Cuore, 29122 Piacenza, Italy; irene.falasconi@unicatt.it (I.F.), alessandra.fontana@unicatt.it (A.F.), guillermo.dusermgarrido@unicatt.it (G.D.G.), laura.principato@unicatt.it (L.P.), giorgia.spigno@unicatt.it (G.S.), lorenzo.morelli@unicatt.it (L.M.)

[2] Biotechnological Research Centre, Università Cattolica del Sacro Cuore, 26100 Cremona, Italy; annalisa.rebecchi@unicatt.it (A.R.), marialuisa.callegari@unicatt.it (M.L.C.),

[†] These authors contributed equally to this work

* Correspondence: vania.patrone@unicatt.it

Supplementary materials

Table S1. Lactic acid bacteria detected during the back-slopping procedure in sorghum sourdoughs. In brackets, the number of strains isolated for each species.

No. of isolates	Identified species	Fermentation days					
		0	1	3	6	8	10
**15**	*Weissella paramesenteroides* (2)	■	■				
6	*Weissella cibaria* (3)	■	■				
39	*Weissella confusa* (5)	■	■				
58	*Pediococcus pentosaceus* (6)		■	■	■	■	■
16	*Pediococcus acidilactici* (1)		■	■	■	■	■
40	*Lactobacillus fermentum* (2)		■	■	■	■	■
4	*Lactococcus taiwanensis* (1)		■				

Table S2. MIC (μg/mL) of antibiotics exhibited by the strains isolated in this study.

Microorganism	Am	Gm	Km	Sm	Em	Cl	Tc	Cm	Nm
W. cibaria (UC4051)	1	1	64	32	1	0.06	4	4	1
W. confusa (UC4052)	2	1	32	16	0.5	0.25	8	4	1
**EFSA cut-off values***	**2**	**16**	**32**	**64**	**1**	**1**	**8**	**4**	**-**
L. fermentum (UC3641)	0.5	1	32	16	0.5	0.12	8	4	2
**EFSA cut-off values**	**2**	**16**	**32**	**64**	**1**	**1**	**8**	**4**	**-**

Antibiotics abbreviations: Am ampicillin, Gm gentamycin, Km kanamycin, Sm streptomycin, Em erythromycin, Cl clindamycin, Tc tetracycline, Cm chloramphenicol, Nm neomycin. *these cut offs are the ones of obligate heterofermentative lactobacilli.

Table S3. General features of the three sequenced genomes, *W. cibaria* UC4051, *W. confusa* UC4052 and *L. fermentum* UC3641, isolated from sorghum sourdough.

	W. cibaria **UC4051**	***W. confusa*** **UC4052**	***L. fermentum*** **UC3641**
Chromosome size (Mbp)	2.44	2.33	1.97
GC content (%)	44.74	44.54	51.70
Scaffolds	56	22	130
tRNAs	81	82	58
rRNAs	9	11	6
CDS	2,293	2,243	1,923
CRISPR	0	0	2
Prophage regions	1	2	0
